# Adolescent Dietary Habit-induced Obstetric and Gynecologic Disease (ADHOGD) as a New Hypothesis—Possible Involvement of Clock System

**DOI:** 10.3390/nu12051294

**Published:** 2020-05-02

**Authors:** Tomoko Fujiwara, Masanori Ono, Michihiro Mieda, Hiroaki Yoshikawa, Rieko Nakata, Takiko Daikoku, Naomi Sekizuka-Kagami, Yoshiko Maida, Hitoshi Ando, Hiroshi Fujiwara

**Affiliations:** 1 Department of Social Work and Life Design, Kyoto Notre Dame University, Kyoto 606-0847, Japan; 2 Department of Obstetrics and Gynecology, Graduate School of Medical Science, Kanazawa University, Kanazawa 920-8640, Japan; masanori@med.kanazawa-u.ac.jp (M.O.); fuji@med.kanazawa-u.ac.jp (H.F.); 3 Department of Integrative Neurophysiology, Graduate School of Medical Science, Kanazawa University, Kanazawa 920-8640, Japan; mieda@med.kanazawa-u.ac.jp; 4 Health Service Center, Kanazawa University, Kanazawa 920-8640, Japan; hiroaki@staff.kanazawa-u.ac.jp; 5 Department of Food Science and Nutrition, Nara Women’s University, Nara 630-8506, Japan; r-nakata@cc.nara-wu.ac.jp; 6 Institute for Experimental Animals, Advanced Science Research Center, Graduate School of Medical Science, Kanazawa University, Kanazawa 920-8640, Japan; tdaikoku@kiea.m.kanazawa-u.ac.jp; 7 Department of Nursing, College of Medical, Pharmaceutical, and Health Sciences, Kanazawa University, Kanazawa 920-8640, Japan; sekky@mhs.mp.kanazawa-u.ac.jp (N.S.-K.); maida@staff.kanazawa-u.ac.jp (Y.M.); 8 Department of Cellular and Molecular Function Analysis, Graduate School of Medical Science, Kanazawa University, Kanazawa 920-8640, Japan; h-ando@med.kanazawa-u.ac.jp

**Keywords:** ADHOGD, adolescent, breakfast skipping, clock gene, dieting, dysmenorrhea, obstetric and gynecological diseases, young adulthood

## Abstract

There are growing concerns that poor dietary behaviors at young ages will increase the future risk of chronic diseases in adulthood. We found that female college students who skipped breakfast had higher incidences of dysmenorrhea and irregular menstruation, suggesting that meal skipping affects ovarian and uterine functions. Since dysmenorrhea is more prevalent in those with a past history of dieting, we proposed a novel concept that inadequate dietary habits in adolescence become a trigger for the subsequent development of organic gynecologic diseases. Since inadequate feeding that was limited during the non-active phase impaired reproductive functions in post-adolescent female rats, we hypothesize that circadian rhythm disorders due to breakfast skipping disrupts the hypothalamic–pituitary–ovarian axis, impairs the reproductive rhythm, and leads to ovarian and uterine dysfunction. To explain how reproductive dysfunction is memorized from adolescence to adulthood, we hypothesize that the peripheral clock system also plays a critical role in the latent progression of reproductive diseases together with the central system, and propose naming this concept “adolescent dietary habit-induced obstetric and gynecologic disease (ADHOGD)”. This theory will contribute to analyzing the etiologies of and developing prophylaxes for female reproductive diseases from novel aspects. In this article, we describe the precise outline of the above hypotheses with the supporting evidence in the literature.

## 1. Introduction

Among young women, insufficient energy intake and inadequate timing of dietary intake have become common nutritional issues in the world [1]. There are growing concerns that poor dietary behaviors in youth will increase the future risk of chronic diseases in adulthood [2]. In general, diet and exercise behaviors in adolescents are influenced by diverse factors such as family, social environment, and peers [3]. In Japan, these inappropriate diet behaviors are partially due to cosmetic reasons [4]. Currently, it is well accepted that adequate calorie restriction improves human health [5,6,7], whereas excess dieting is also known to induce several gynecological disorders such as dysmenorrhea and irregular menstruation [8,9]. It was also reported that dietary behaviors are closely related to the onset or management of polycystic ovary syndrome [10,11] and hypothalamic amenorrhea [12,13], suggesting the close relationship between gynecological disorders and dietary habits [14]. Furthermore, it was proposed that gynecologic diseases such as endometriosis, which are frequently manifested by dysmenorrhea, can latently develop with a common modern dietary lifestyle [15]. 

Based on these backgrounds, we found that female college students who skipped breakfast had a significantly higher incidence of dysmenorrhea than those who ate breakfast, according to a questionnaire-based investigation [16]. In addition, when we strictly limited the definition of a regular menstrual cycle to a 26–32-day cycle, a significantly higher incidence of irregular menstruation was observed in the group that skipped breakfast [17], suggesting that skipping breakfast disrupts ovarian and uterine functions in young women [9].

From these findings, we propose a novel concept that disorders of the central clock system due to breakfast skipping disrupt the hypothalamic-pituitary-ovarian axis, impair the reproductive rhythm, and lead to ovarian and uterine dysfunction. We also hypothesize that the peripheral clock system plays a critical role in the latent progression of reproductive diseases. In this article, we precisely present the outline of the above hypothesis, showing the supporting evidence in the literature.

## 2. Breakfast Skipping and Menstrual Disorders

Meal-skipping rates are high during young adulthood [18]. Currently, adolescence is defined as between 10 and 19 years old, while youth is between 15 and 24 years old [19]. Accordingly, these periods can be divided into early adolescence (10–14 years), late adolescence (15–19 years), and young adulthood (20–24 years) [1]. Japanese college students (19–22 years old) are in the post-adolescent stage, which is between adolescence and young adulthood. Neurologically, distinct maturation of the human brain was observed after adolescence by magnetic resonance imaging (MRI) [20,21]. Consequently, we suggest that the post-adolescent stage is also important for maturation of the reproductive function [4]. In Japan, when starting college life, high rates of students start living alone. Accordingly, this post-adolescent stage should be understood as a critical period when daily behaviors, including dietary habits, can become poor.

Our longitudinal questionnaire-based study subsequently confirmed the positive relationship between skipping breakfast and the incidence of dysmenorrhea [17]. Later, several other studies reported similar findings [22,23,24]. Helwa et al. demonstrated that skipping breakfast was the strongest predictor of moderate/severe dysmenorrhea among Palestinian female university students (*n* = 956) [23]. Another study also showed that skipping breakfast was an associated risk factor of primary dysmenorrhea among Chinese female university students (*n* = 4606) [24]. Although one study reported no significant correlation between breakfast skipping and dysmenorrhea, it may be because the authors classified participants having breakfast one to six times per week in the normal range of breakfast eating [25]. On the other hand, Gagua et al. reported that meal skipping (twice or less meal intake per day) was one of the most important risk factors for dysmenorrhea in 2890 women aged 14–20 years who were randomly selected in Georgia [26]. 

## 3. Breakfast Skipping and Other Disorders

Currently, meal skipping is considered to increase future risks of various chronic metabolic diseases [2,18,27]. Skipping breakfast is associated with other adverse dietary habits such as high intakes of fast and processed foods. Female college students with these adverse dietary habits demonstrated a high incidence of dysmenorrhea, confirming that dietary habits influence the reproductive function [28]. Skipping breakfast is also significantly correlated with poor physical conditions and mental health [17,29]. Despite the absence of a significant difference in the body mass index (BMI), the high incidence of a self-perception of poor general health was observed in the group that skipped breakfast. In contrast, the groups with high intakes of fast and processed foods did not complain of poor general health [28]. Although the precise mechanisms are unclear, the presence of starvation caused by breakfast skipping at the initial stage of daily activity may explain these differences [17].

Female college students who skipped breakfast also showed a tendency to have constipation [16,30,31,32,33]. In general, food intake induces bowel movement via the parasympathetic nerve pathway, being effective especially just after waking-up [34,35,36]. Since skipping breakfast decreases stimulations of digestive organs in the morning when social activity becomes high, a high incidence of constipation may correspond to a rhythmic discrepancy between central and peripheral automatic nerve systems [30]. Considering the possibility that constipation influences the environment of the pelvic cavity, the relationship between constipation and female genital organic disorders should be examined in the future [30,34].

## 4. Past History of Dieting and Dysmenorrhea

In general, body-related teasing is known to induce body dissatisfaction and influence dieting behaviors in adolescents [37]. Additionally, female adolescents are currently inclined to lose body weight for cosmetic reasons in Japan. Our study showed that more than 60% of female college students used diet control in order to reduce body weight, and about 40% of students had undergone dietary restriction in adolescence [4]. Since the majority of students who dieted showed normal or under the normal range of BMI values [4], their choice of dietary restriction is partially due to the discrepancy between their BMI and self-recognition of appropriate body weight. 

In accordance with previous reports [38,39], we observed that dieting female college students had a higher incidence of irregular menstruation. It was also reported that dieting is associated with dysmenorrhea [9,39]. Notably, when we classified the population into students currently on a diet and those with a history of dieting in adolescence, students with a history of dieting showed a significant increase in dysmenorrhea despite the absence of a current increase in irregular menses or decrease in BMI. Namely, the frequency of irregular menstruation is mainly increased in young women who are currently on a diet, while the intensity of dysmenorrhea is high in those with a history of dieting in adolescence [4]. This tendency was observed throughout the six years examined [13]. These findings suggest that dietary habits in adolescence have long-lasting effects on the reproductive function in young women. On the other hand, diet was reported to be closely associated with meal skipping [2,40]. Based on these findings, we proposed a novel concept that adverse dietary habits in adolescence become a trigger for the subsequent development of organic gynecologic diseases, including endometriosis, which are characterized by dysmenorrhea [4,17].

## 5. Adolescent Dietary Habit-Induced Obstetrics and Gynecologic Disease (ADHOGD)

**Figure 1:**
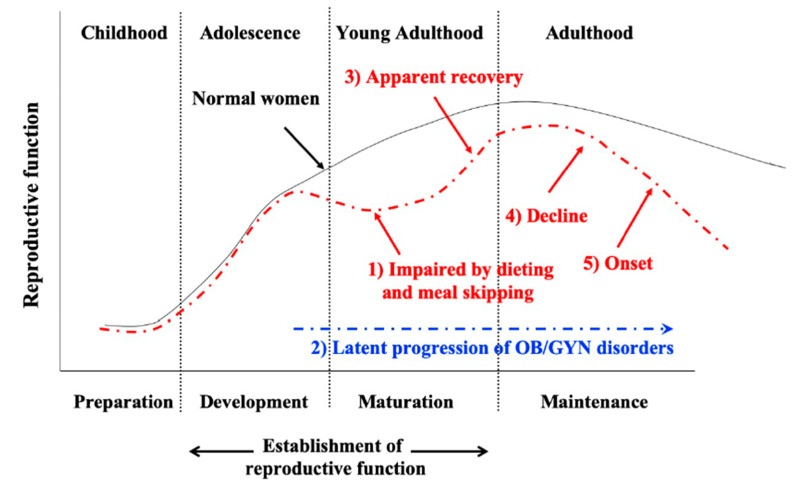
Concept of adolescent dietary habit-induced obstetric and gynecologic disease (ADHOGD). Adverse dietary habits, such as dieting and breakfast skipping during adolescence and adulthood, impair development and maturation of the reproductive function (1), which induces the latent progression of obstetric and gynecologic disorders (2). Although apparent recovery is achieved after correcting adverse eating habits (3), the reproductive function is precociously declined (4), which later leads to the onset of obstetrics and gynecologic diseases (5).

Currently, the hypothesis of developmental origins of health and disease (DOHaD) is causing marked concern throughout the world [41]. This concept was initially called “fetal origins of adult disease” [42], proposing that exposure to certain environmental influences such as undernutrition during critical periods of development and growth may determine the onset of human diseases in adulthood [43]. The DOHaD theory focuses on prenatal and perinatal stages as window periods when predictive adaptive responses can occur in the presence of environmental influences [44]. In contrast, considering that reproductive organs extensively develop, grow, and mature during adolescence and adulthood, these stages are critical periods for establishment of the female reproductive function. Consequently, as shown in Figure 1, we previously proposed that adverse dietary habits, such as dieting and breakfast skipping during adolescence and adulthood, impair development and maturation of the reproductive function (1), which induces latent progression of obstetrics and gynecologic disorders (2). Although recovery is achieved after correcting adverse eating habits (3), the reproductive function declines (4), and this leads to the latter onset of obstetrics and gynecologic diseases (5) [4,17]. To make it more comprehensive, we propose naming this concept “adolescent dietary habit-induced obstetric and gynecologic disease (ADHOGD)”.

## 6. Mechanism of Breakfast Skipping-Induced Dysmenorrhea

Primary dysmenorrhea is mainly caused by abnormal uterine contraction [45,46], which is usually derived from hormonal disorders in ovarian function [47]. In the adolescent stage, functional immaturity in production of ovarian sex steroid hormones stimulates local production of prostaglandins E2 and F2 and leukotrienes that induce excessive uterine contractions with pain during menstruation [46,48]. Clinically, when mature women have dysmenorrhea without apparent signs of ovarian dysfunction such as irregular menstruation, several organic disorders should be suspected [49]. Endometriosis is one of the most important gynecological diseases manifested as dysmenorrhea [50,51], which occasionally needs laparoscopic diagnosis and treatment [52]. Although the exact etiology remains unclear, retrograde menstrual effluent into the peritoneal cavity caused by abnormal uterine contraction is considered to induce ectopic implantation of endometriotic lesions [53]. The affected lesions gradually spread throughout the pelvic cavity through periodic ovulation and menstruation, resulting in severe tissue adhesion, pelvic pain, and infertility at a reproductive age [54]. Thus, ovarian dysfunction and abnormal uterine contraction are essential factors for dysmenorrhea.

Skipping breakfast interferes with the start of the active phase during the circadian rhythm that is regulated by the central clock system [55]. Since both food intake and the light/dark cycle are the main regulators of circadian rhythms [56,57], skipping breakfast can lead to changes in light stimulation within the central clock system. Consequently, we speculate that this confliction of circadian rhythm by meal skipping affects the hypothalamic-pituitary-ovarian axis, impairs reproductive rhythm, and leads to ovarian dysfunction [17,58]. To investigate this, we examined the relationship between the timing of food intake during the circadian cycle and reproductive function during the estrus cycle, using young female rats [58]. In the daytime-fed group (fed only in the non-active phase), the frequency and number of ovulations were significantly decreased compared with those in the control and nighttime-fed group (fed only in the active phase), indicating that the timing of food intake during the daytime or nighttime is an important factor regulating the function of the hypothalamic-pituitary-ovarian axis in post-adolescent female rats [58,59].

## 7. Possible Involvement of the Central Clock System in Reproductive Dysfunction 

Recently, the relationship between the circadian rhythm and the reproductive function has become a general concern [60]. Circadian rhythms are produced by synchronized transcriptional oscillators and associated molecules that are encoded by clock genes such as *brain and muscle aryl hydrocarbon receptor nuclear translocator like protein 1 (BMAL1)*, *Clock*, *Periods (Per)*, and *Cryptochrome (CRY)* [61]. Light and dark cycles entrain the hypothalamic suprachiasmatic nucleus, and these neurons act as master pacemakers for circadian behavioral rhythms in mammals [55]. Currently, the close relationship between the hypothalamic suprachiasmatic nucleus (master clock) and kisspeptin-producing neurons, which is a central regulator of the hypothalamic-pituitary-ovarian axis, has been demonstrated [62,63]. In addition, it was reported that dietary energy balance influences the activity of kisspeptin neurons, which may suppress fertility in women with under-nutritional conditions [64]. Considering that reproductive rhythms are newly developed and matured from adolescence to young adulthood, we must pay attention to the potentially marked adverse effects of desynchronization between the central (master clock) and peripheral brain clock (kisspeptin neuron) systems on the normal establishment of the hypothalamic-pituitary-ovarian axis. 

Transgenic mice with clock gene mutations were reported to show disrupted reproductive functions such as in ovarian steroidogenesis [65], estrous cyclicity, and the maintenance of pregnancy [66]. *Bmal1*-knockout mice were also reported to be infertile and show implantation failure with a reduction of ovarian functions [67,68]. Accordingly, we consider that differences between daytime-fed and nighttime-fed groups are partially derived from disturbance of the clock system [58].

## 8. Possible Involvement of the Peripheral Clock System in Uterine Dysfunction

Recently, we found that pregnant women who had a history of dysmenorrhea in young adulthood showed a high incidence of hypertensive disorders of pregnancy (HDP), whereas dysmenorrhea just prior to pregnancy was not significantly correlated with the onset of HDP. HDP is derived from the abnormal placental formation due to inadequate trophoblastic invasion into the maternal uterus [69]. Accordingly, these findings suggest that certain uterine disorders latently continue even after dysmenorrhea has improved and manifest again as functional abnormalities during pregnancy. This is consistent with the ADHOGD theory. However, before theoretically accepting the above mechanism, it is necessary to explain how uterine dysfunction is memorized from adolescence to adulthood. To answer this, we paid attention to the peripheral clock system in the uterus together with the central system.

Peripheral oscillators, which are mainly regulated by daily food intake cycles, operate in most peripheral organs [70]. Circadian clock genes such as *Per1-3*, *CRY1-2*, *BMAL1*, and *Clock* were demonstrated to be expressed in the uterus and oviduct in rodents [71,72]. Very recently, it was reported that the silencing of *BMAL1* gene expression in human endometrial stromal cells attenuated the in vitro decidualization of stromal cells [73]. Notably, *BMAL1* silencing also impaired the ability of decidual cells to regulate trophoblast invasion, which can impair adequate placental formation. To suggest the involvement of clock dysfunction in obstetric disorders, the authors additionally demonstrated that *BMAL1* gene expression was downregulated in the endometrium of women who experience recurrent spontaneous abortion [73]. To support the above speculation, our preliminary experiments using uterus-specific *Bmal1*-deficient mice demonstrated that uterine *Bmal1* gene expression was essential to maintain a successful pregnancy. On the other hand, a recent study showed that feeding at an unusual time of day (inactive phase) desynchronizes peripheral clocks, causing obesity and metabolic disorders in adult mice [74]. In addition, a breakfast-skipping mouse model showed that the timing of feeding regulated peripheral clock gene expression in the liver [75]. We also observed that clock gene expressions in the murine uterus can be affected by the timing of food intake.

**Figure 2:**
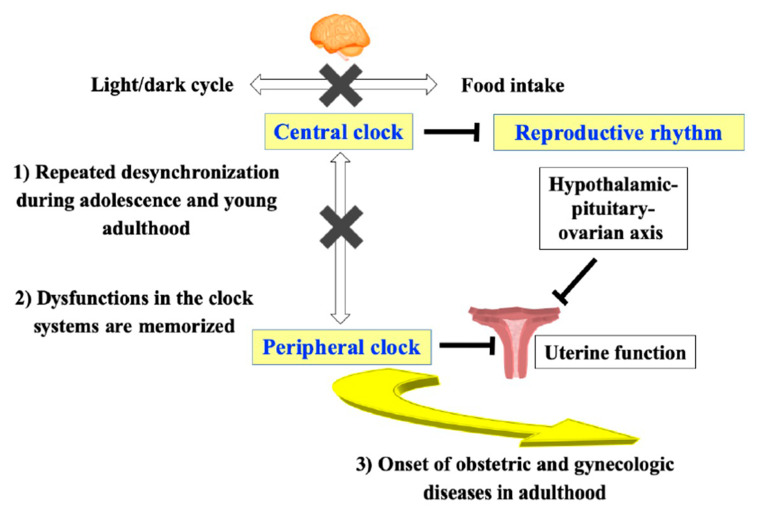
The involvement of central and peripheral clock systems in ADHOGD. Adverse dietary habits continue to desynchronize central and peripheral clocks during the critical period, from adolescence to young adulthood (1). This functional disturbance in the clock systems is memorized in the hypothalamic-pituitary-ovarian axis and the uterus from the developing to mature stages (2). This will lead to the onset of obstetric and gynecologic diseases in adulthood (3).

Based on these findings, we hypothesized that the peripheral clock system in the uterus also plays a critical role in ADHOGD. It should be noted that the uterus extensively develops, grows, and matures during adolescence and young adulthood from both morphological and functional aspects [76,77]. Therefore, if food intake at an unusual time of day continues to desynchronize central and peripheral clocks during these critical periods, this functional disturbance in the clock system can be memorized in the uterus from the developing to mature stages, and this will lead to the onset of obstetric and gynecologic diseases in adulthood (Figure 2). 

## 9. Conclusions

Young women who skip breakfast show significantly higher incidences of dysmenorrhea and irregular menstruation, suggesting that meal skipping affects ovarian and uterine functions. Since dysmenorrhea becomes more manifested in those with a past history of dieting, we proposed a novel concept whereby inadequate dietary habits in adolescence become a trigger for the subsequent development of organic gynecologic diseases. As a possible mechanism, we speculate that confliction of circadian rhythm by meal skipping affects the hypothalamic-pituitary-ovarian axis, impairs the reproductive rhythm, and leads to ovarian and uterine dysfunction. We hypothesize that the peripheral clock system in the uterus also plays a critical role in the latent progression of reproductive diseases and propose naming this concept “adolescent dietary habit-induced obstetric and gynecologic disease (ADHOGD)”. This concept will contribute to analyzing the etiologies of and developing prophylaxes for female reproductive diseases such as polycystic ovary syndrome, hypothalamic amenorrhea, endometriosis, infertility, preterm labor, and so on from novel aspects. This hypothesis may change the focus from therapeutic to prophylactic and from dietary content to dietary timing in the management of gynecologic disorders in young women. Further investigation together with developing new methods is recommended to test the hypothesis in the future. 

## Abbreviations

Adolescent dietary habit-induced obstetric and gynecologic disease	ADHOGD
Body mass index	BMI
Brain and muscle aryl hydrocarbon receptor nuclear translocator like protein 1	BMAL1
Cryptochrome	CRY2
Developmental origins of health and disease	DOHaD
Hypertensive disorders of pregnancy	HDP
Magnetic resonance imaging	MRI
Periods	Per
